# Improvements in Interdisciplinary Communication Following the Implementation of a Standardized Handoff Curriculum: SAFETIPS (Statistics, Assessment, Focused Plan, Pertinent Exam findings, to Dos, If/Thens, Pointers/Pitfalls, and Severity of Illness)

**DOI:** 10.7759/cureus.56384

**Published:** 2024-03-18

**Authors:** Shaefali Shandilya, Justen M Aprile

**Affiliations:** 1 Pediatrics, Penn State Health Children's Hospital, Hershey, USA

**Keywords:** safe patient handover, undergraduate and graduate medical education, interprofessional education and collaboration, communication in healthcare, multi-disciplinary team

## Abstract

Background

Handoffs between medical providers serve a crucial patient safety function. While most published literature on the topic studies the handover process among physicians, robust literature is available on interdisciplinary medical communication. Little is known about the downstream effects of effective physician handover on subsequent physician and nursing interactions.

Objective

Our objective was to implement a handoff curriculum, SAFETIPS (Statistics, Assessment, Focused plan, pertinent Exam findings, To dos, If/thens, Pointers/pitfalls, and Severity of illness), for pediatric residents and to investigate its impact on nurses’ perceptions of resident preparedness, efficiency, and competency.

Methods

Nurses were asked to score residents in five domains and describe the frequency of nurse-to-resident and resident-to-nurse interruptions. The survey was distributed before and after the SAFETIPS introduction.

Results

Statistical analysis revealed significant post-intervention mean score increases of one full point in four categories, namely organization and efficiency, communication, content, and clinical judgment. The percentage of nurses using the term “reasonable/relevant” to describe interactions with residents significantly increased from 45% to 76% (p = 0.004). The percentage of nurses reporting that residents gave “unsure response[s],” made decisions that differed from nurses’ decisions, and made decisions without family/parental interests significantly decreased by 31 (p = 0.004), 22 (p = 0.034), and 30 (p = 0.002) percentage points, respectively.

Conclusion

The introduction of a structured handoff curriculum significantly improves communication among residents. This is then associated with improved interactions between residents and nurses.

## Introduction

The physician handoff is a critical component of patient care, and shift-to-shift handoffs are a fixture of hospital medicine [1]. In a 2017 report, the Joint Commission reiterated the importance of using “standardize[d] tools and methods,” specifically mnemonic devices, to “structure handoff communication and help team members perform handoffs more consistently” [2]. In 2011, the Accreditation Council for Graduate Medical Education (ACGME) introduced new restrictions on intern work hours, limiting single shifts to 16 hours and work weeks to 80 hours. Consequently, the number of patient care transitions, or handoffs, increased [3]. In 2017, the ACGME revised their nearly six-year-old regulations, increasing work-hour caps on all residents, interns, and seniors alike to 28 hour-shifts after finding that “the disruption of team-based care and supervisory systems […] had a significant negative impact on […] the effectiveness of care delivery of the team as a whole” [4]. Before this study, the pediatric residents at our hospital did not utilize a standardized handoff tool. In light of both the Joint Commission’s and ACGME’s findings and recommendations, we deemed it particularly important to identify and improve points of communication breakdown between patient care teams in the handoff process within our pediatric residency program. This study, a subset of a larger study examining the impact of a structured handover curriculum on resident and physician handover/communication, sought to investigate whether introducing a handover curriculum to pediatric residents would impact nurses’ perceptions of those residents' preparedness and decision-making.

Roughly 80% of serious medical errors stem from miscommunication during patient transfers of care [5]. Increased handoff frequency leads to an increase in the potential number of opportunities for medical errors to occur. Outcome-dependent medical error, or error associated with adverse patient outcomes, is the primary focus of studies examining transitions of care and medical error. Numerous studies have examined outcome-dependent error as it relates to physician handoff, using mortality as an outcome measure. A 2015 study of over 23,000 patients found that end-of-service resident handoffs were still significantly associated with an increase in all-cause hospital mortality, despite modest improvements following the 2011 decision to reduce duty hours [6].

Multiple handoff mnemonic devices have been described in the literature [7]. One of the earliest and most commonly used handoff tools is SBAR (Situation, Background, Assessment, and Recommendation) [8]. The SBAR mnemonic is somewhat limited when more in-depth information is required for a complex patient [9]. Other proposed handoff devices include the SIGNOUT and IPASS the BATON (Introduction, Patient, Assessment, Situation, Safety Concerns, Background, Actions, Timing, Ownership, Next) methods; both demonstrated efficacy but were limited by the length of the acronym and minor redundancies in the included information [9-11]. The I-PASS (Illness severity, Patient summary, Action list, Situational awareness and contingency planning, and Synthesis or read-back) study aimed to address all of these limitations and is one of the most impactful studies on handoffs since 2011 [9].

Despite the considerable success of I-PASS, investigators continue to identify opportunities for growth in the quality of handoffs. In fact, not only are structured curricula crucial, but studies continue to emphasize the importance of communication among differing care team providers (clinicians, nursing for example) [12-17]. Even beyond the mnemonic device, an overarching limitation of handoff tools was the lack of faculty ownership and interest in resident learning [18]. A study of intern handoffs highlighted other persistent limitations, notably the lack of face-to-face communication, opportunities to ask questions, private handoff locations, accompanying written documentation, non-distracting environments, and minimization of interruptions [19]. The actual mnemonic utilized can vary, as long as it is part of a structured curriculum and contains the essential elements of patient information identified across handover curricula [20]. When adapting a comprehensive handoff curriculum for our institution, we decided that instead of including more information under each individual letter within the IPASS acronym, a slightly longer mnemonic device would allow for a greater systematic approach to each section of handoff, with a resultant decrease in inter-resident variability. The SAFETIPS mnemonic allowed us to expand on the components of I-PASS and better tailor our sign-out tool to the pediatric residents and their training program [21,22-24]. The mnemonic stands for Statistics, Assessment, Focused plan, pertinent Exam findings, To dos, If/thens, Pointers/pitfalls, and Severity of illness.

The ACGME, in their 2016 Clinical Learning Environment Report, highlighted the importance of interdisciplinary collaboration in systems-based improvement efforts [22]. Despite the numerous studies on physician handoffs, information regarding the relationship between handoffs and interdisciplinary communication among members of the medical team is scarce [12,15]. We hypothesized that a structured written and verbal handoff curriculum would improve resident efficiency and competency at handoff, which would thus improve nurses’ perceptions of resident preparedness and decision-making. This investigation represents an innovative, inter-professional approach to assessing the efficacy of a handoff curriculum as the clinical relationship between nursing staff and residents is significantly impacted by the quality of communication between these staff members.

## Materials and methods

SAFETIPS curriculum

As a quality improvement project, this study was deemed exempt from our medical center’s Institutional Review Board. The initial phase of the study spanned approximately three years and included all pediatric and medicine-pediatric residents (73 in total) at our medical center. This larger, longitudinal study similarly observed improved communication scores both when residents observed peers and when independent observers observed the handover process. The SAFETIPS tool was initially adapted by the principal investigator (PI) at a previous institution [23]. After minor modifications, this curriculum was introduced to the pediatric residents for use during handoffs. All team members, including attending physicians and medical students, were included in the implementation process.

The PI collaborated with Cerner® system administrators to create a SAFETIPS-formatted handoff document that was then embedded within Powerchart®, our existing electronic medical record (EMR) system. In the months immediately before implementation, the PI held three educational sessions with residents and faculty members to present the SAFETIPS acronym and handoff method. Approximately one week after the final education session, the new handoff curriculum went into effect. Each verbal handoff was accompanied by a written component, aligning with the SAFETIPS mnemonic. With the implementation of SAFETIPS, senior residents were also now required to be present at intern sign-out.

Pre-implementation phase

Using REDCap software, a nursing evaluation tool was developed, which was modeled on a validated Likert scale created specifically to evaluate handoffs [24]. The evaluation was distributed to the inpatient pediatric nursing staff, consisting of approximately 140 nurses covering 54 beds on the general acute care floor, intermediate care unit, and hematology-oncology unit. These units comprise all med/surg inpatient pediatric beds excluding the intensive care unit. The survey asked nurses to reflect on their interactions with pediatric residents, specifically to assess a resident’s preparedness to discuss patient care, and ability to clarify information and answer questions appropriately.

**Figure 1:**
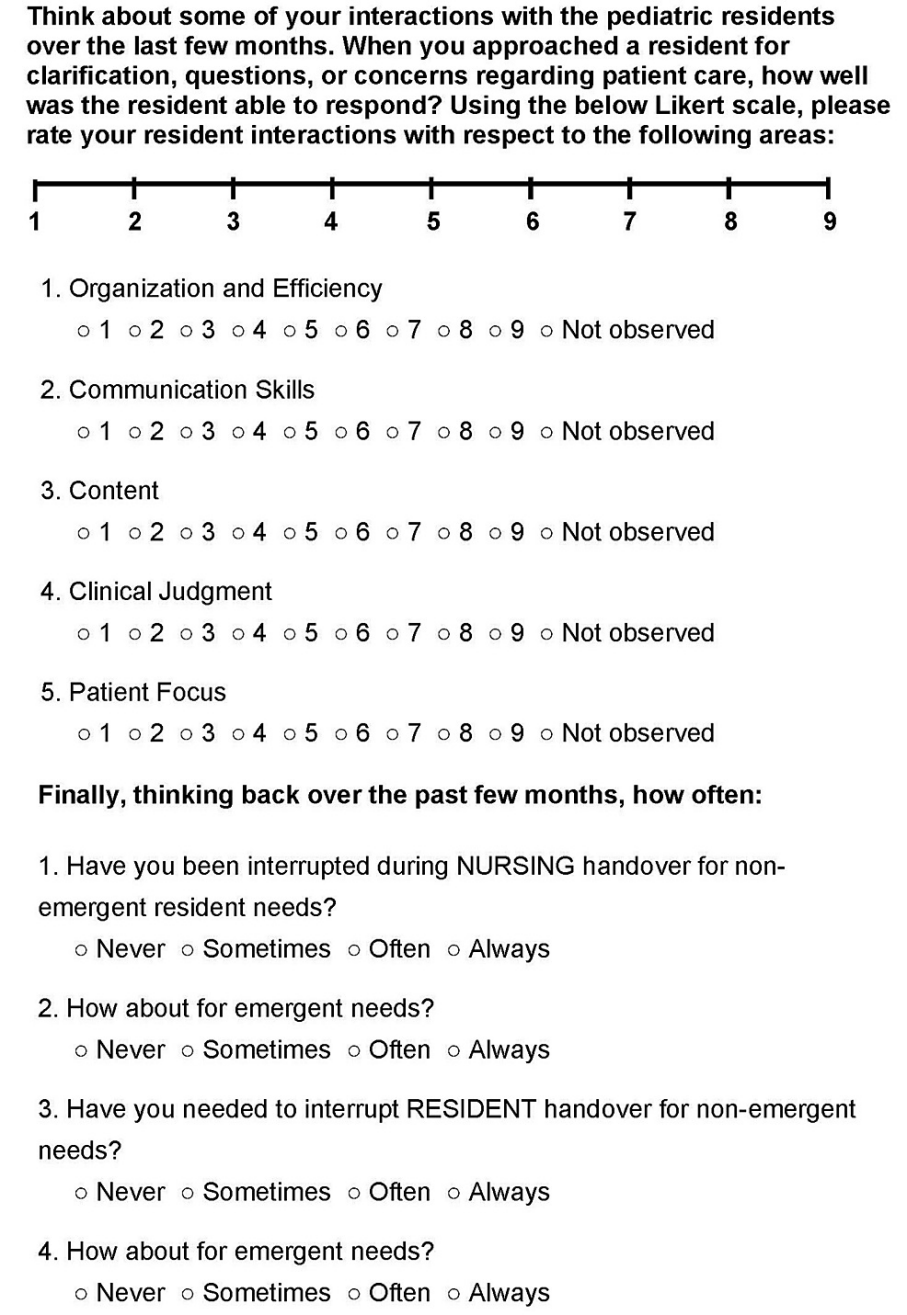
REDCap survey distributed to pediatric nurses

**Figure 2:**
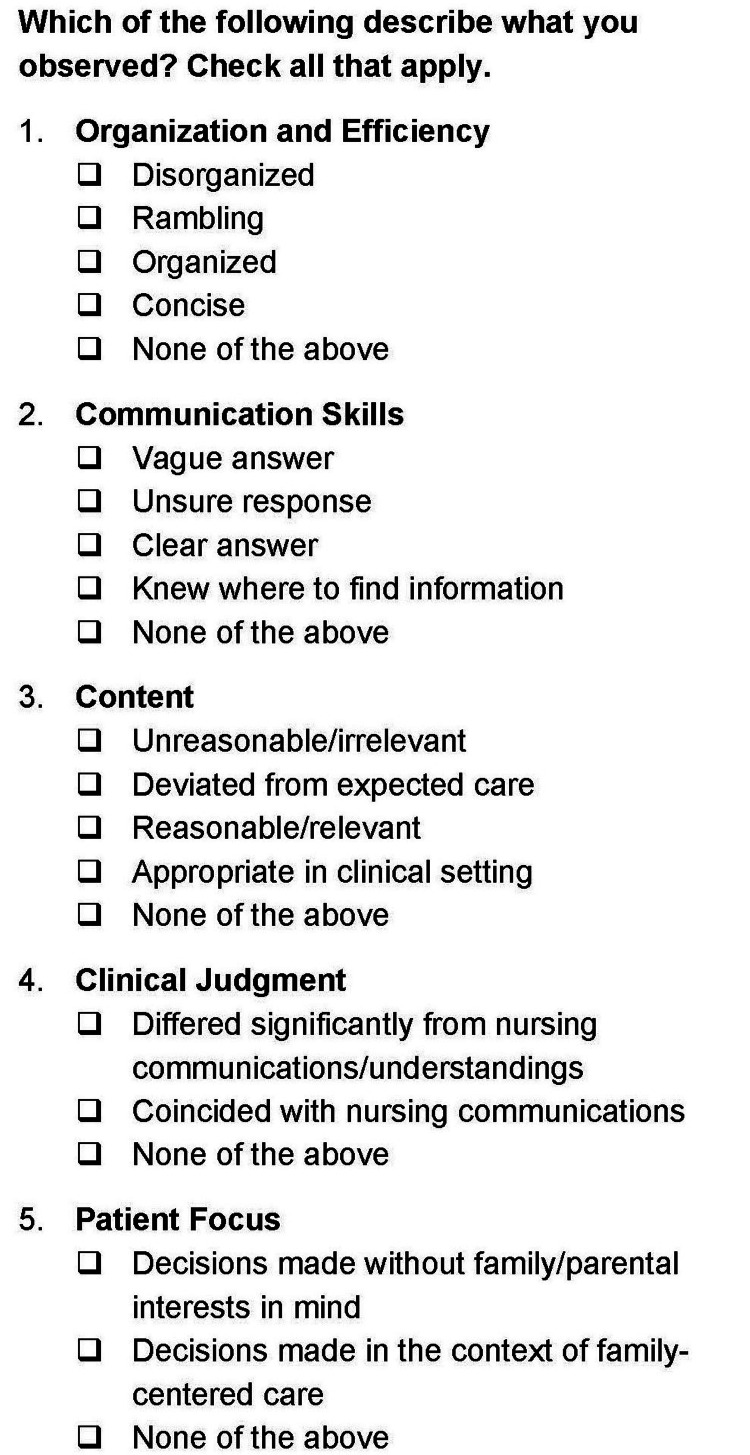
Specific descriptors for each of the five survey parameters in Figure 1

Nurses evaluated residents on the following parameters: organization and efficiency, communication skills, content of interactions, clinical judgment, and patient focus (Figure 1). Under each of these categories, nurses also had the option to select descriptors to characterize the interactions more specifically (Figure 2). For instance, they could describe the residents’ organization and efficiency using the following terms: “organized,” “disorganized,” “rambling,” and “concise.”

Nurses were also asked to assess the number of times they were interrupted by residents for emergent and non-emergent needs during their nursing handoff; similarly, they were asked to report the frequency with which they interrupted resident handoff for emergent and non-emergent needs (Table 1). Nursing staff had three weeks to complete the pre-implementation survey. After initial evaluations were completed, the SAFETIPS model was implemented.

**Table 1:** Interruptions during nursing and resident handoffs for emergent and non-emergent needs No statistically significant changes were observed in any category. SAFETIPS: Statistics, Assessment, Focused plan, pertinent Exam findings, To dos, If/thens, Pointers/pitfalls, and Severity of illness.

	Pre-SAFETIPS (n = 58)	Post-SAFETIPS	p-value
How often have you:		(n = 37)	
Been interrupted during NURSING handover for non-emergent resident needs?			0.057
Never/Sometimes	31 (53.4%)	27 (73%)	
Often/Always	27 (46.6%)	10 (27%)	
Been interrupted during NURSING handover for emergent resident needs?			0.207
Never/Sometimes	48 (82.8%)	34 (91.9%)	
Often/Always	10 (17.2%)	3 (8.1%)	
Needed to interrupt RESIDENT handover for non-emergent needs?			0.248
Never/Sometimes	53 (91.4%)	36 (97.3%)	
Often/Always	5 (8.6%)	1 (2.7%)	
Needed to interrupt RESIDENT handover for emergent needs?			0.883
Never/Sometimes	46 (82.1%)	30 (83.3%)	
Often/Always	10 (17.9%)	6 (16.7%)	
No response	2 (0.03%)	1 (0.03%)	

Post-implementation phase

Four months after the SAFETIPS curriculum was introduced, post-implementation evaluations were distributed to the nursing staff. Evaluation forms were identical for the pre- and post-implementation periods.

Data collection and analysis

All survey responses and evaluations were recorded within REDCap. For statistical analysis, responses were categorized into Likert scale responses or binary responses. For Likert scale responses, two-sample Wilcoxon tests were used. For binary outcomes, two-sample chi-squared tests were used. For the survey questions regarding interruptions during emergent or non-emergent situations,, variables were dichotomized as never/sometimes versus often/always and then treated as binary outcomes.

## Results

Of the 140 nurses surveyed, 58 (41.4%) responded before implementation, and 37 (26.4%) responded after implementation. Table 2 shows the Likert responses related to nurse-resident interactions with respect to resident organization, communication, content, clinical judgment, and patient focus in the pre- and post-implementation periods. Nurses were not required to submit responses for every field of the survey, which is reflected in the differences in n values for each category. The smaller number of responses post-implementation was noted, and still, all five categories saw increases in score after SAFETIPS implementation, and statistically significant (p < 0.05) increases in the score were observed in four out of five categories: organization, communication, content, and clinical judgment.

**Table 2:** Nurses’ perceptions of residents based on Likert responses before and after SAFETIPS implementation p-values < 0.05 are statistically significant. SAFETIPS: Statistics, Assessment, Focused plan, pertinent Exam findings, To dos, If/thens, Pointers/pitfalls, and Severity of illness.

	Pre-SAFETIPS	Post-SAFETIPS	p-value
Organization and efficiency			0.003
n	56	33	
Mean (SD)	5.7 (1.49)	6.7 (1.42)	
Median	6	7	
Range	8-Feb	9-Apr	
Communication			0.005
n	57	34	
Mean (SD)	5.7 (1.63)	6.7 (1.30)	
Median	6	7	
Range	9-Feb	9-Apr	
Content			0.045
n	56	33	
Mean (SD)	6.1 (1.58)	6.8 (1.23)	
Median	6	7	
Range	9-Mar	9-Apr	
Clinical judgment			0.014
n	58	34	
Mean (SD)	5.8 (1.59)	6.7 (1.31)	
Median	6	7	
Range	8-Feb	9-Apr	
Patient focus			0.113
n	57	34	
Mean (SD)	6.3 (1.67)	6.9 (1.67)	
Median	7	7	
Range	9-Feb	9-Feb	

**Figure 3:**
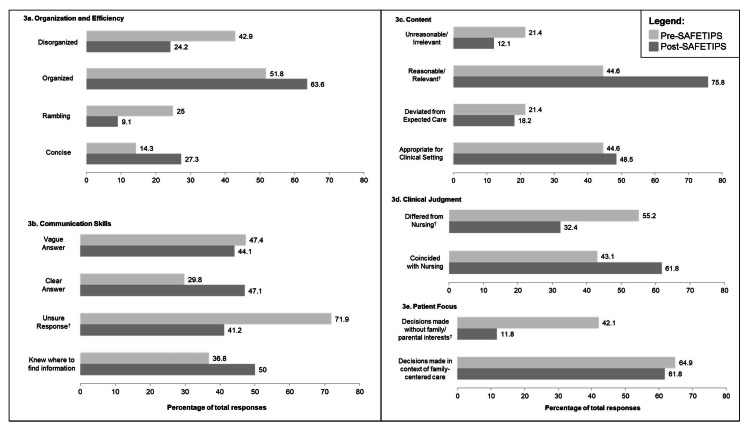
(a-e) Trends in the nurses’ use of specific descriptors by category, before and after SAFETIPS † indicates that there was a significant (p < 0.05) increase or decrease in the use of the descriptor after SAFETIPS was introduced. SAFETIPS: Statistics, Assessment, Focused plan, pertinent Exam findings, To dos, If/thens, Pointers/pitfalls, and Severity of illness.

Nurses had the option to further describe their interactions by category (Figure 2). Again, this was contingent on them assigning a Likert score to that category, which is reflected in the different n values. Figure 3 summarizes these responses, pre- and post-SAFETIPS. Under the “Organization and Efficiency” category (Figure 3, Panel a), a higher percentage of nurses described residents as “disorganized” and “rambling” before SAFETIPS, but after implementation, a higher percentage of nurses used the terms “organized” and “concise.” Under the “Communication Skills” category (Figure 3, Panel b), a greater percentage of nurses noted that residents had “clear answer[s]” and “knew where to find information” following SAFETIPS implementation. Notably, there was a significant decrease (p = 0.004) in the percentage of nurses who used the term “unsure response” to describe residents’ communication after SAFETIPS was introduced. Under the “Content” category (Figure 3, Panel c), there was a significant increase (p = 0.004) in the percentage of nurses who described residents’ communication as “reasonable/relevant” after SAFETIPS. Under the “Clinical Judgment” category (Figure 3, Panel d), there was a significant decrease (p = 0.034) in the percentage of nurses who believed that residents’ decisions “differed from nursing” after SAFETIPS was introduced. Finally, in the “Patient Focus” category (Figure 3, Panel e), there was a significant decrease (p = 0.002) in the percentage of nurses who reported that resident decisions were “made without family/parental interests” after SAFETIPS was introduced.

The third portion of the survey asked nurses to estimate how frequently they interrupted resident handoff for emergent and non-emergent needs and how frequently residents interrupted nursing handoff for emergent and non-emergent needs. Response options were “never,” “sometimes,” “often,” and “always.” For statistical analysis, responses were dichotomized into never/sometimes and often/always and treated as binary outcomes (Table 1). For frequency of interruptions, both by nurses and by residents, there was an increase in the percentages of “never” and “sometimes” responses and a decrease in percentages of “often” or “always” responses in all categories from pre- to post-implementation. However, none of these trends was statistically significant.

## Discussion

This study sought to investigate whether introducing the SAFETIPS curriculum to pediatric residents would affect nurses’ perceptions of residents. We demonstrate that the introduction of a standardized handoff curriculum for our group of residents was correlated with a measurable improvement in the nurses’ perceptions of residents during their interactions. This improvement was reflected in the significant increases in scores in four out of five categories of the nursing survey. Moreover, the data on nurses’ use of descriptors conveyed a larger trend. After SAFETIPS was integrated into the residency, the overall percentage of nurses who used negative descriptors (e.g., “disorganized” and “unreasonable/irrelevant”) decreased, while the percentage of nurses who used positive descriptors (e.g., “concise” and “reasonable/relevant”) increased in all five categories. Notably, the significant decrease in the percentage of nurses who reported that resident decisions differed from their own demonstrates an increased concordance between nurses and residents in clinical decision-making, likely fostered by improved communication. Improved communication may also account for the significant decrease in the percentage of nurses who believed that residents were making decisions “without family/parental interests.” Finally, although not statistically significant, the overall reduction in reported interruptions, both by residents and nurses, signifies that improved inter-professional communication may have reduced the need for urgent clarifications or inquiries regarding patient care. It may also imply that nurses and residents increasingly respected each other’s handoffs as protected time and thus sought to minimize interruptions. The study underscores the significance of not only focusing on the clinician to improve handover, and therefore patient care, but also studying and strengthening the communication among large, interdisciplinary care teams when trying to impart meaningful, and lasting, change [12-15,25].

The implementation of SAFETIPS aimed to provide residents with a standardized format for handoff to help ensure that they included all essential patient information without missing key points or providing extraneous details. By incorporating both written and verbal components, the curriculum encouraged residents to evaluate their patients within the SAFETIPS framework while using the EMR and communicating face-to-face. Together, our findings suggest that introducing SAFETIPS into the pediatric residency program provided a structure to help residents improve their organization, efficiency, clarity, and ability to communicate their clinical decision-making to the extent that the nursing staff was able to observe these improvements. A broader goal of this study was to translate the improvement in resident communication to an improvement in inter-professional communication. By eliciting nurses’ perspectives, this study represents a unique approach to evaluating a handoff curriculum.

While the aforementioned studies have investigated the impact of implementing standardized handoffs on residents, patient outcomes, and medical errors, to date, there have been few studies on the impact of resident handoffs on interdisciplinary communication with nurses [16,17]. As medical care is increasingly delivered in a team-based format, facilitating clear communication between members of the medical team, particularly house staff and nurses, becomes especially important [14]. Underscoring the cruciality of this notion is expertly cited by Pattabi et al. as “The nurse-physician relationship set the tone for unit culture and hence all measures need to be taken to develop a collegial and collaborative relationship. One of the most important determinants of patient safety and quality of care offered by the hospitals will depend upon the nurse-physician relationship of the individual units and unit culture” [26]. Sezgin and Bektas, along with a similar article by Bender et al., highlighted simulation-based education programs/workshops to foster communication among team members, especially interventions initiated at the student level [13,14]. All of these interventions were shown to improve collaboration, teamwork, and patient outcomes [13,14]. Farhadie et al. also commented on the relationship between improved communication and improved job satisfaction, a well-established contributor to healthcare provider burnout [27]. Taken together, not only do structured curricula play a key aspect in improving communication, but such educational measures also work to improve collaboration, teamwork, and collegiality with the singular intention of improving patient care and outcomes.

Limitations

Three limitations were encountered in this study. First, nurses who responded to the survey were not required to identify themselves. Thus, nurses may have submitted the survey at both time points (pre- and post-SAFETIPS), but they cannot be accounted for, and their responses cannot be analyzed in pairs. All data was thus analyzed using two-sample methods that assume independence between the groups. The Wilcoxon test, a non-parametric alternative to the t-test, was used, with the consequence that p-values that are just barely within the threshold of significance (i.e., just <0.05) may not reflect a robust statistical correlation. Second, this study utilized a subjective survey as its primary means of data collection, and thus, it is possible that external biases unrelated to the implementation of SAFETIPS had an influence on nurses’ perceptions of residents over time. Third, the overall response rate was low for both the pre- and post-implementation surveys.

## Conclusions

Our study offers evidence that improved physician communication can have a significant rippling effect on other members of the medical care team. While a formalized handover curriculum improved resident communications, it likewise significantly impacted nurses’ perceptions of resident preparedness, efficiency, and competency. Based on our findings, investigators seeking to study physician handoff may benefit from considering other members of the medical team, including nurses, when evaluating the efficacy and impact of a standardized handoff curriculum.
